# Large bowel obstruction due to sesame seed bezoar: a case report

**DOI:** 10.1186/1752-1947-1-159

**Published:** 2007-11-29

**Authors:** Aidan G Shaw, Oliver Peacock, Jonathan N Lund, Gillian M Tierney, Mike Larvin, William Speake

**Affiliations:** 1 Department of Gastrointestinal Surgery, University of Nottingham, Derby, UK; 2 Department of Colorectal Surgery, Derby City General Hospital, Derby, UK

## Abstract

We report a case of a 79 year old man with a known benign anastomotic stricture presenting with large bowel obstruction. At laparotomy the obstruction was found to be caused by a large sesame seed bezoar. Seed bezoars are well known to cause impaction in the rectum but have never been previously reported to cause large bowel obstruction. We recommend that patients with known large bowel strictures should be advised not to eat seeds as this could ultimately lead to obstruction, ischaemia or perforation.

## Introduction

A bezoar is a mass of swallowed foreign indigestible material found within the gastrointestinal tract. They have been known to occur in animals and man for centuries. Classification depends on content – phytobezoars (plant fibres), trichobezoars (hair), and lactobezoars (milk curds). They may occur in combinations like trichophytobezoars, and can result from virtually anything capable of forming concretions within the gastrointestinal tract, including medications [1]. They are most commonly found in the stomach, rarely found in the colon and may lead to anorexia, weight loss, bleeding, obstruction, or perforation of the alimentary tract [2]. Gastrointestinal bezoars have constituted a relatively common clinical reality since the introduction of truncal vagotomy associated with drainage or gastric resection in the treatment of gastroduodenal peptic ulcer [3].

A variety of bezoars have been reported to have caused small bowel obstruction – medication [4,5], cotton [6], dry fruit [7], furniture cushion foam [8] and fruit and vegetable fibres [9]. No literature could be found of large bowel obstruction secondary to bezoars.

## Case presentation

**Figure 1:**
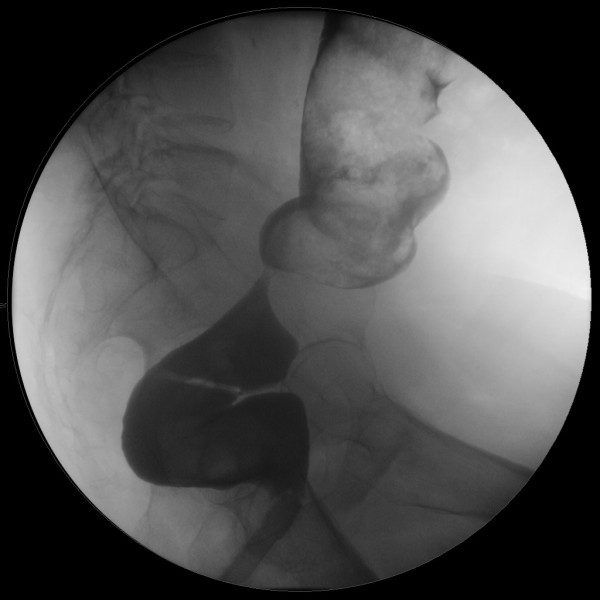
Double contrast barium enema demonstrating anastomotic stricture prior to dilatation, two years before the obstructive episode.

A 79 year old man presented four years previously to the Urologists with recurrent urinary tract infections and pneumaturia. Cystoscopy revealed a colo-vesical fistula and he was subsequently referred to the colorectal surgeons. Barium enema demonstrated a stricture in the sigmoid colon and computerised tomography revealed an inflammatory mass in the sigmoid colon with communication into the bladder. He underwent a sigmoid colectomy, with histology showing diverticular disease. Two years later he developed colicky lower abdominal pain and a change in bowel frequency. Barium enema and subsequent sigmoidoscopy revealed an anastomotic stricture, with biopsies demonstrating benign disease (Figure 1). The stricture was successfully dilated by balloon, and his symptoms resolved.

**Figure 2:**
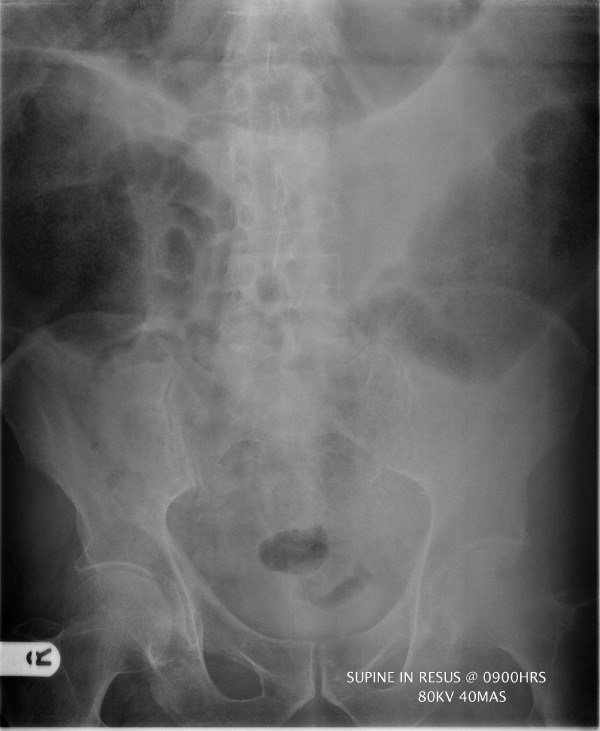
AXR demonstrating large bowel obstruction to the level of the rectosigmoid junction.

A further two years later he was admitted to Accident and Emergency with generalised abdominal pain, distension, and vomiting. On arrival, he was found to be hypotensive, tachycardic and peripherally shut down. Abdominal examination revealed distension and generalised abdominal tenderness with localised peritonitis over the right side of the abdomen. Blood tests revealed a marked metabolic acidosis (ph 7.23 pCO_2 _4.0 kPa p0_2 _9.1 kPa Base excess -13.4 mmol/L Lactate 3.7 mmol/L HCO_3 _12.6 mmol/L) and a high white cell count (WCC 23 × 10^9^/L). Abdominal X-Ray (AXR), performed in the resuscitation room, demonstrated distension of the colon to the level of the rectosigmoid junction with a point of transition in this region (Figure 2). Chest X-Ray revealed no free air under the diaphragm. After a period of resuscitation, it was decided to proceed to laparotomy as the patient had signs of ischemic bowel.

Operative findings were of a dilated large bowel to the level of the anastomosis, three areas of ischaemic colon (caecum, transverse and sigmoid regions), extensive small bowel adhesions and a dense fibrous pelvis. A subtotal colectomy and ileostomy was performed and on resection it was found that the stricture was patent with a good luminal diameter. The obstruction was found to have been caused by a large bezoar of sesame seeds which had impacted within and above the lumen of the stricture.

The gentleman has made an uneventful recovery and on further questioning he recalls regularly eating nuts and seeds as part of his healthy diet.

## Discussion

Seed bezoars in the rectum have been considered an uncommon cause of faecal impaction in adults. Sunflower seed impaction in the rectum has been frequently reported, with all cases requiring either manual disimpaction under general anaesthesia or endoscopic management for resolution of their symptoms [10,11]. One study in Israel found that seed bezoars in the rectum were the most common cause of faecal impaction requiring hospitalization with all 30 patients needing digital disimpaction under general anaesthesia. The conclusion was that the consumption of seeds with shell fragments or fruits containing many seeds (such as the prickly pear) should be accompanied by the awareness that large quantities may cause faecal impaction [12].

In our case, it is difficult to say where the seeds formed into a bezoar. The most likely sites would be in the small bowel or the caecum, from where it travelled and lodged at the anastomotic stricture.

## Conclusion

Here we have reported the first case of large bowel obstruction secondary to a bezoar. The learning point should be that patients with known large bowel strictures should be advised not to eat seeds as this could ultimately lead to obstruction, ischaemia or perforation.

## Competing interests

The author(s) declare that they have no competing interests.

## Authors' contributions

AS wrote the manuscript;

ML performed the laparotomy with AS

GT took over the care and follow up of the patient

JL, OP, ML and WS reviewed the literature

All authors, contributed intellectual content, have read and approved the final manuscript

## Consent

Consent was obtained from the patient for publication of the study and the X-rays.
